# A comparison of lower limb EMG and ground reaction forces between barefoot and shod gait in participants with diabetic neuropathic and healthy controls

**DOI:** 10.1186/1471-2474-11-24

**Published:** 2010-02-03

**Authors:** Isabel CN Sacco, Paula MH Akashi, Ewald M Hennig

**Affiliations:** 1 University of São Paulo, School of Medicine, Physical Therapy, Speech and Occupational Therapy Department, São Paulo, Brazil; 2 University of Duisburg-Essen, Biomechanics Laboratory, Essen, Germany

## Abstract

**Background:**

It is known that when barefoot, gait biomechanics of diabetic neuropathic patients differ from non-diabetic individuals. However, it is still unknown whether these biomechanical changes are also present during shod gait which is clinically advised for these patients. This study investigated the effect of the participants own shoes on gait biomechanics in diabetic neuropathic individuals compared to barefoot gait patterns and healthy controls.

**Methods:**

Ground reaction forces and lower limb EMG activities were analyzed in 21 non-diabetic adults (50.9 ± 7.3 yr, 24.3 ± 2.6 kg/m^2^) and 24 diabetic neuropathic participants (55.2 ± 7.9 yr, 27.0 ± 4.4 kg/m^2^). EMG patterns of vastus lateralis, lateral gastrocnemius and tibialis anterior, along with the vertical and antero-posterior ground reaction forces were studied during shod and barefoot gait.

**Results:**

Regardless of the disease, walking with shoes promoted an increase in the first peak vertical force and the peak horizontal propulsive force. Diabetic individuals had a delay in the lateral gastrocnemius EMG activity with no delay in the vastus lateralis. They also demonstrated a higher peak horizontal braking force walking with shoes compared to barefoot. Diabetic participants also had a smaller second peak vertical force in shod gait and a delay in the vastus lateralis EMG activity in barefoot gait compared to controls.

**Conclusions:**

The change in plantar sensory information that occurs when wearing shoes revealed a different motor strategy in diabetic individuals. Walking with shoes did not attenuate vertical forces in either group. Though changes in motor strategy were apparent, the biomechanical did not support the argument that the use of shoes contributes to altered motor responses during gait.

## Background

Many studies have been performed examining changes in gait biomechanics of diabetic neuropathic populations [1-7]. The previous described gait alterations are suspected to predispose diabetic neuropathic patients to foot ulcer formation.

The main changes in gait biomechanics caused by the presence of peripheral diabetic neuropathy include higher plantar pressures [8-11], alterations in spatio-temporal patterns [1,4,12-15] and greater stance phase time [2,5,6]. Kinetic parameters changes during gait have also been observed and include modified ground reaction forces and moments of force [1,2,4,12,16-18], as well as decreased and delayed lower limb muscle activity. In particular, the vastus lateralis, tibialis anterior, and gastrocnemius have been the most affected by the neuropathy progression [3,5-7]. All of these alterations may play an important role in foot ulcer formation, in addition to other autonomic complications[19,20].

All of the previously discussed gait alterations have been studied during barefoot gait, which does not represent the usual daily living locomotion condition, especially among diabetic individuals. However, it is still unknown if these biomechanical changes are also present during shod gait, which is highly recommended for diabetic patients to prevent foot ulceration. It is known that the use of shoes changes the sensory input to the motor control system [21-24] and it also may alter the already reduced plantar sensitivity caused by the diabetic neuropathy, which could modify the motor responses and adjustments of these participants during load accommodation. In other words, there may be additional biomechanical changes during shod gait that would attempt to protect the foot and lower limbs against overloads. The biomechanical adjustments during shod gait in patients with diabetic neuropathy are not yet clear.

Although the use of footwear is considered an important factor to prevent diabetic foot ulcers (due to its effect on plantar pressure redistribution), there are no studies that compares biomechanical gait patterns in diabetic individuals with and without shoes. These parameters have already been compared in patients with knee osteoarthritis [25], after knee ligament reconstruction [26] and in runners [27], some of them favour the barefoot condition for load attenuation of the injured joint [25,26,28,29].

There are several mechanisms by which footwear may influence lower limb biomechanics. It has been suggested that less ankle range of motion caused by the use of shoes may partially block the foot rollover process that could alter the rocker action of the foot and ankle necessary for normal function and biomechanics of the lower limbs [30]. One of the consequences of reduced range of motion during gait is a loss of the ankle's eccentric control occurring from heel strike to flat foot phase that can cause an alteration in the shock absorption mechanism and may increase loads applied to the foot of diabetic patients [31].

Other findings suggest that during barefoot gait the longitudinal plantar arch seems to be higher, potential causing a more enhanced load accommodation [32,33]. The higher plantar arch observed when walking barefoot could be due to a motor strategy attempting to change the plantar architecture for better shock absorption. Adequate afferent information, from the plantar surface of the foot during contact with the floor, is an essential element for these foot adaptations and may be altered when wearing shoes [27,32,33]. In the shod condition, the plantar arch loses its full capacity for load attenuation [27] due to the alterations in afferent information and the lack of malleability caused by the shoes. This loss of load attenuation capacity can be related to the high injury frequency in shod locomotion conditions [27,33].

This study aimed to comparing ground reaction forces and lower limb muscle activity (EMG) during gait with and without the use of regular shoes worn on a daily basis between diabetic neuropathic individuals and healthy controls. It was hypothesized that walking with shoes influences the plantar sensory information available, resulting in altered ground reaction forces and delayed muscle activity.

## Methods

### Subjects

Forty-five adult volunteers participated in this study and were divided into two groups: a control group (CG) composed of 21 healthy non-diabetic participants (age = 50.9 ± 7.3 yr, BMI = 24.3 ± 2.6 kg/m^2^) and 24 diabetic neuropathic participants (DG) (age = 55.2 ± 7.9 yr, BMI = 27.0 ± 4.4 kg/m^2^). Ethics approval was obtained from the Local Ethics Committee. The volunteers provided written informed consent to participate in the study. All neuropathic participants were diagnosed by a physician. Inclusionary criteria consisted of: at least 5 years post-onset of Type 2 diabetes, a minimum of two plantar areas with deficits on tactile sensitivity by not recognizing a 10 g monofilament [10,34,35] and a score higher than 6 in the Michigan Neuropathy Screening Instrument - questionnaire (MNSI-q) for symptoms related to the diabetic neuropathy [36]. The exclusion criteria adopted for both groups included an age over 65, due to alterations in gait simply caused by aging, partial or total amputation, orthopedic disorders of the lower limbs, pain during the data collection, use of any assistive devices for walking (walking sticks/canes) and the presence of plantar ulcers at the time of the evaluation.

### Procedure

EMG activity of right vastus lateralis (VL), lateral gastrocnemius (GL) and tibialis anterior (TA) muscles and ground reaction forces were collected simultaneously during the stance phase of both barefoot and shod gait. Participants were requested to walk both barefoot and using their habitual shoes (the shoes they used most frequently during daily activities) at a self-selected cadence across a 10 m walkway with a force plate embedded in its center. EMG activity was sampled at 1000 Hz during three trials in each gait condition using the EMG System do Brasil (Sao José dos Campos, Brazil). The bipolar surface electrodes were placed according to SENIAM recommendations [37]. Electrode diameter was 10 mm with an inter-electrode distance of 22 mm. After shaving and cleaning each area with alcohol, electrodes were attached to the skin using both Transpore adhesive tape (3 M, Sumaré, Brazil) and an elastic band (Tensor, Cotia, Brazil). The vertical and horizontal antero-posterior ground reaction forces (GRF) were collected using an AMTI force plate (Watertown, MA, USA) at 1000 Hz.

To reduce variability, the type of shoes were controlled and matched for both groups, so that the groups wore proportionally the same kind of shoes: sport shoes (30%), loafers (30%), sandals (25%) and dress shoes (15%). None of the participants used customized orthopedic/therapeutic shoes on a daily basis.

### Numerical and Statistical analysis

For the EMG data, first the DC offsets were removed, the signals were then full-wave rectified, and passed through a zero lag 4^th ^order Butterworth low-pass filter with a cutoff frequency of 5 Hz. Finally the EMG signals were time normalized to 100% of the stance phase, which was determined using the GRF. The GRF data were processed using a zero lag low-pass Butterworth 4^th ^order filter with a cutoff frequency of 100 Hz and then normalized to each subject's body weight and also time normalized to 100% of the stance phase.

All data were processed and variables were calculated in a custom-written program using Matlab v.7.1 (MathWorks, Inc.). The EMG variables for the TA, VL and LG muscles were time to peak EMG during the stance phase of gait. The vertical GRF variables consisted of the first and second peaks vertical force and the minimum value between the two peaks. The antero-posterior GRF variables consisted of the peak braking horizontal force and the peak propulsive force.

Levene test and Shapiro-Wilks test were used to assess each variables homocedasticity and that each one was normally distributed. Statistical tests included a 2 (group) × 2 (condition) analysis of variance (ANOVA) to assess the EMG and GRF variables. The Newman-Keuls post hoc test was used following each of the ANOVAs (α = 0.05; 0.05 < α < 0.10 = trend to significant different variables).

## Results

When examining stance phase time the results demonstrated that there was no significant difference between the groups for both gait conditions (CG × DG shod p = 0.10; barefoot p = 0.33) This was performed to verify that both groups presented with similar gait cadence, once differences in stance phase could be caused by different cadences adopted by the subjects.

**Figure 1:**
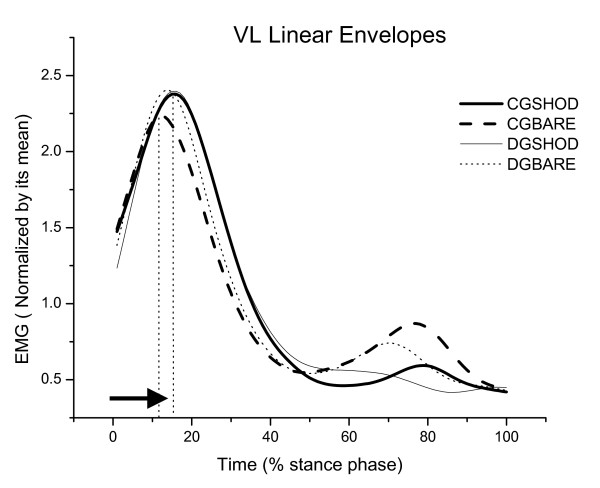
**Mean of the linear envelopes of the right vastus lateralis muscle (VL), normalized according to the mean of the control (CG) and diabetic (DG) groups during shod and barefoot gait**. Note the delayed peak in CG shod, DG barefoot and shod gait conditions.

**Figure 2:**
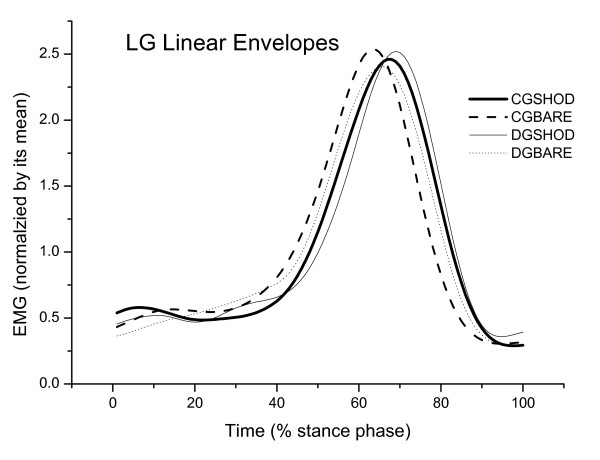
**Mean of the linear envelopes of the right lateral gastrocnemius muscle (GL), normalized according to the mean of the Control (CG) and diabetic (DG) groups during shod and barefoot gait**.

**Figure 3:**
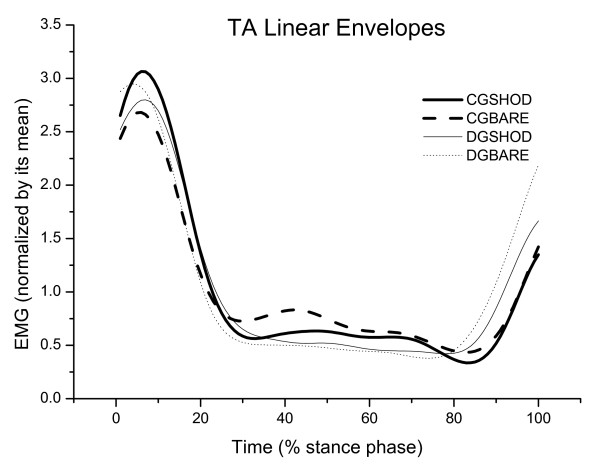
**Mean of the linear envelopes of the right tibialis anterior muscle (TA), normalized according to the mean of the Control (CG) and diabetic (DG) groups during shod and barefoot gait**.

The results for time to peak EMG are presented in Table 1, Figure 1, Figure 2 and Figure 3 for both conditions (shod and barefoot gait).

**Table 1:** Mean (and standard deviation) of Vastus lateralis, Lateral gastrocnemius and Tibialis anterior time of peak occurrence of control group (CG) and diabetic group (DG), during the stance phase in both condition group: barefoot and shod gait.

				ANOVA			Effect size	
**Time of peak occurrence (%)**	**Condition**	**CG (n = 21)**	**DG (n = 24)**	**Group**	**Condition**	**Group × Condition**	**DG relative to CG (95% CI)**	**Shod relative to Barefoot (95% CI)**

**Vastus lateralis**	**Shod**	15.47 ± 4.27	15.35 ± 3.71	**F = 5.12**	**F = 10.68**	F = 3.74	-0.120 (- 2.809 to 2.569)	2.960 (1.305 to 4.614)
	**Barefoot**	10.76 ± 2.81	14.14 ± 2.35	**p = 0.030**	**p = 0.002**	p = 0.06	3.380 (1.639 to 5.121)	

**Lateral gastrocnemius**	**Shod**	66.41 ± 4.31	68.23 ± 3.84	F = 1.29	**F = 10.98**	F = 0.20	-1.820 (-0.813 to 4.453)	2.590 (0.528 to 4.652)
	**Barefoot**	64.17 ± 3.92	65.29 ± 5.35	p = 0.263	**p = 0.002**	p = 0.654	1.120 (- 2.033 to 4.273)	

**Tibialis anterior**	**Shod**	6.52 ± 3.08	6.58 ± 2.82	F = 0.20	F = 3.36	F = 0.06	0.060 (-1.925 to 2.045)	1.020 (-0.216 to 2.256)
	**Barefoot**	5.46 ± 2.36	5.61 ± 2.39	p = 0.655	p = 0.06	p = 0.804	0.150 (-1.447 to 1.746)	

Effect size (mean difference (95% CI)) is also provided for each variable.

**Figure 4:**
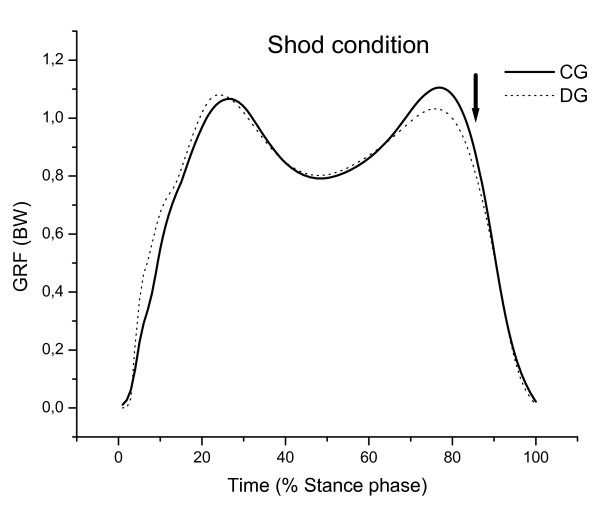
**Mean of vertical GRF curves in shod gait condition normalized according to each participant's body weight of the control (CG) and diabetic (DG) groups**. Note the lower second vertical peak in DG.

**Figure 5:**
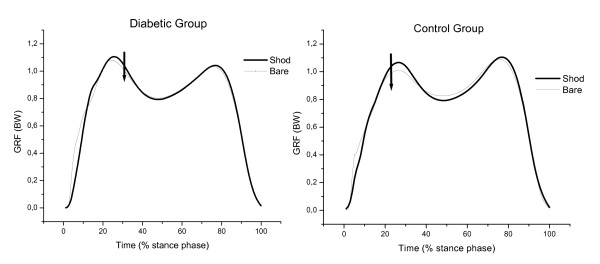
**Mean of vertical GRF curves in shod and barefoot gait condition normalized according to each participant's body weight of the diabetic (DG) and control group (CG)**. Note the lower first vertical peak in barefoot condition in both groups.

**Figure 6:**
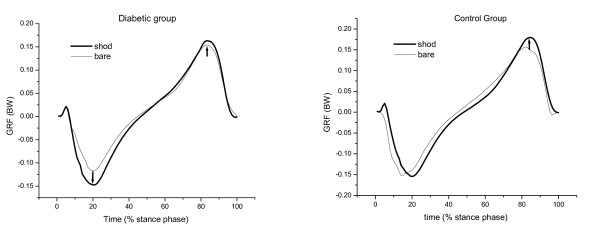
**Mean of braking and propulsion GRF curves in shod and barefoot gait condition normalized according to each participant's body weight of the diabetic (DG) and control group (CG)**. Note the higher braking force in shod condition in both groups, and the higher propulsion force in shod condition in CG.

The results for vertical and horizontal GRF are presented in Table 2, Figure 4, Figure 5 and Figure 6 for both groups during each of the gait conditions.

**Table 2:** Mean (and standard deviation) of frst, second and minimum value between these vertical GRF peaks, braking and propulsion GRF peaks of control group (CG) and diabetic group (DG), during the stance phase in both condition group: barefoot and shod gait.

				ANOVA			Effect size	
**GRF (times bodyweight)**	**Condition**	**CG (n = 21)**	**DG (n = 24)**	**Group**	**Condition**	**Group × Condition**	**DG relative to CG (95% CI)**	**Shod relative to Barefoot (95% CI)**

**First Vertical Peak**	**Shod**	1.09 ± 0.09	1.12 ± 0.07	F = 1.29	**F = 22.42**	F = 0.11	0.030 (- 0.024 to 0.084)	0.040 (0.004 to 0.075)
	**Barefoot**	1.04 ± 0.09	1.08 ± 0.06	p = 0.262	**p < 0.001**	p = 0.742	0.040 (-0.011 to 0.091)	

**Second Vertical Peak**	**Shod**	1.11 ± 0.07	1.05 ± 0.05	**F = 6.68**	F = 3.15	F = 0.80	- 0.060 (- 0.101 to - 0.019)	0.0200 (-0.011 to 0.051)
	**Barefoot**	1.09 ± 0.07	1.04 ± 0.07	**p = 0.014**	p = 0.085	p = 0.378	- 0.050 (- 0.097 to - 0.003)	

**Minimum value between peaks**	**Shod**	0.77 ± 0.07	0.76 ± 0.07	F = 0.64	**F = 8.97**	F = 0.002	- 0.010 (- 0.057 to 0.037)	-0.030(-0.061 to 0.001
	**Barefoot**	0.81 ± 0.08	0.81 ± 0.07	p = 0.428	**p = 0.005**	p = 0.967	0.000 (- 0.051 to 0.051)	

**Braking force**	**Shod**	-0.142 ± 0.04	-0.152 ± 0.05	F = 0.02	**F = 14.58**	F = 2.81	- 0.010 (- 0.040 to 0.020)	-0.020 (-0.032 to -0.008)
	**Barefoot**	-0.131 ± 0.02	-0.125 ± 0.04	p = 0.873	**p < 0.001**	p = 0.103	0.006 (- 0.015 to 0.027)	

**Propulsion force**	**Shod**	0.178 ± 0.02*	0.168 ± 0.03	F = 0.64	**F = 25.18**	F = 0.68	- 0.010 (- 0.027 to 0.007)	0.020 (0.011 to 0.029)
	Barefoot	0.155 ± 0.02*	0.152 ± 0.03	p = 0.428	**p < 0.001**	p = 0.412	- 0.003 (- 0.020 to 0.014)	

Effect size (mean difference (95% CI)) is also provided for each variable.

Once there was no significant Group × Condition interaction effects in the statistical analysis, in the results are presented first the Group effect then the Condition effect.

### Group effect

There was a significant group effect in the VL time to peak EMG [F(1,32) = 5.12; p = 0.03] and in the second peak vertical GRF [F(1,32) = 6.68; p = 0.01]. The VL muscular activity showed a marginally significant difference in group × condition interaction effect [F(1,32) = 3.74, p = 0.06]. Diabetic participants presented with a delayed VL activity comparing to controls during barefoot gait (p = 0.005) which was found to be 3.5% of the stance phase (23 ms). Diabetic participants also demonstrated a 6% of body weight reduction during the shod gait condition for the second peak vertical GRF compared to controls (p = 0.01).

### Condition effect

There was a significant effect for condition in the VL time to peak EMG [F(1,32) = 10.68; p = 0.002], GL [F(1,32) = 10.98; p = 0.002] and a strong trend for TA time to peak EMG [F(1,32) = 3.63; p = 0.06]. During shod gait, there was a significant delay in the LG activity among diabetic participants (p = 0.03, post hoc test) and a delay in the VL activity among controls (p = 0.004, post hoc test). The delay in the LG activity was 3% of the stance phase among diabetic participants (approximately 21 ms) and the delay in the VL activity was 5% among controls (approximately 35.2 ms).

There was a significant effect for condition in the first peak vertical GRF [F(1,32) = 22.42; p < 0.001], peak braking force [F(1,32) = 14.58; p < 0.001] and peak propulsive force [F(1,32) = 25.18; p < 0.001]. The first peak vertical GRF was significantly higher in both groups during the shod gait condition. The difference between both gait conditions among diabetic participants was found to be 5% of body weight higher (p = 0.01, post hoc test) and among the controls was found to be 4% of body weight (p = 0.003, post hoc test). Peak braking and propulsive forces were also higher in diabetic participants during shod gait (braking force was 3% of body weight, p = 0.002; propulsive force was 1.5% of body weight, p = 0.01, post hoc test). Among controls, only the peak propulsive force was higher during shod gait (2% above body weight, p = 0.002, post hoc test).

## Discussion

The present study investigated whether ground reaction forces and lower limb EMG activity were altered during gait with and without the use of regular shoes worn on a daily basis between diabetic neuropathic individuals and healthy controls. To date, the authors are unaware of any current literature that supports the role of shoes in modifying muscular responses or altering GRF during diabetic neuropathic gait. The main results support our initial hypotheses that walking with shoes used on a daily basis, delays lower limb muscle activity and alters vertical and horizontal ground reaction forces. However, diabetic participants demonstrated different motor adjustments during shod gait compared to controls since their muscle activation was already delayed in barefoot gait.

The results of this study showed that when walking with shoes, both control and diabetic participants presented with a higher vertical peak GRF at initial ground contact and a higher propulsive force. The smaller vertical peak GRF and propulsive force during barefoot gait could be caused by a more precautious gait adopted by both groups. The results from the first vertical peak during shod gait contradict the popular belief that the use of shoes attenuates external loads during walking, particularly in diabetic neuropathic subjects. Hennig et al. (1994) have also contradicted these beliefs. They observed that the first vertical peak GRF was lower when the participants wore harder shoes, based on a self perception cushioning scale. Diverti et al. (2005) analyzed ground reaction forces during barefoot and shod running and also found lower values during the barefoot condition, which is typically considered the harder condition. The authors have suggested that barefoot running leads to a reduction of the initial impact peak due to a neural-mechanical adaptation in order to reduce high mechanical stress occurring during repetitive steps.

Shakoor and Block (2006) found lower knee forces during barefoot gait in participants with osteoarthritis compared to shod gait (habitual shoes). The authors suggested that walking barefoot may increase proprioceptive inputs from skin contact with the ground, leading to a more precautious gait pattern. In this study, the diabetic group had a pre-existing afferent deficit in the plantar surface of the foot. Walking barefoot may have increased the proprioceptive inputs causing an enhanced efferent pattern, which may have decreased the values of the first vertical peak in an attempt to reduce joint loads.

The second vertical peak was smaller in the diabetic group compared to the control group when wearing shoes. However, this difference was not observed during barefoot gait. Since no changes in GL muscle activity was observed between groups, the use of shoes may be partially blocking the foot roll over process and restricting normal ankle motion, which is already altered in the diabetic population [38-42]. This could modify the foot rollover mechanism during propulsion phase, that corresponds to the phase in which the second peak occurs and may have influenced its magnitude.

There were no differences in antero-posterior GRF peaks between groups in either gait condition. However, when comparing gait conditions the diabetic group had a higher horizontal braking force when wearing shoes, which is similar to the higher vertical peak during the initial stance phase. The diabetic individuals may be adopting a strategy to increase the sensitive inputs by increasing the force when they contact their heel to the floor. Also, the diabetics presented a delayed VL activity compared to controls, which may cause a higher braking force.

The alterations in sensorimotor control, due to the use of shoes, were different in controls compared to diabetic participants. The neuromuscular system will generate responses according to the afferent sensory information caused by mechanical loads placed on the foot. Considering the reduced plantar sensitivity in the diabetic neuropathic participants, it is possible that the muscle recruitment strategy to attenuate joint loads is altered in these patients. Due to the decreased plantar sensitivity, changes in EMG and external loads would be expected [2,3,5-7] and were observed in the present study.

Apparently, the interaction between the use of shoes and altered afferent sensory information has lead to adjustments in efferent muscular responses, which modified the lower limb biomechanical kinetic parameters. It was not only the use of shoes that caused these changes, nor the sensorial deficit isolated, but their interaction.

It has previously been shown that changing the afferent information of the plantar surface during gait can alter lower limb muscle activity [43]. Therefore, a delayed muscular activation pattern during shod gait in all participants was expected, especially at initial ground contact when the afferent information is even more altered with shoes [21-23]. The expected neuromuscular delays are thought to be related to the muscles involved in shock attenuation, which include the quadriceps femoris and tibialis anterior [44]. In the present study, the healthy participants had a delay in the VL at initial ground contact with shoes compared to barefoot. The diabetic group did not have a delay in the VL activity at initial ground contact between the shod and barefoot conditions. However, the diabetic group kept the same pattern of VL activation when wearing shoes compared to walking barefoot, they had a delayed VL activation compared to the control group. During the shod condition, only the diabetic group had a delay of the GL activity, which may demonstrate an altered sensorimotor strategy when dealing with different plantar sensorial information wearing shoes. This demonstrates that the decreased plantar sensitivity caused by the disease may have a greater effect on the neuromuscular control than the afferent changes seen with the use of shoes.

There was no effect of the disease or the gait condition on TA activity. This finding is in agreement with previous results that did not find any delays in TA [6,7]. However, the current results also conflict with other studies [3,5]. Sacco and Amadio (2003) found subtle delays in the TA EMG activity of diabetics (around 6% of the stance phase), which justify further studies to investigate the temporal organization of the TA activation in the stance phase.

There are some limitations to the present study, which should be identified. Although shoe type was attempted to be controlled and matched between groups, it may have added some variability to the EMG and GRF data. A universal shoe was not used due to the need for each participant to adapt to a new pair of shoes, which may interfere with the biomechanical data.

The present results reveal that the pattern of muscle activation in the stance phase of diabetic individuals is still a matter of debate, as put forward by Allet et al. (2008)[45]. The study of the EMG time series and its relation to recruitment firing rate will contribute to elucidate the locomotor pattern in neuropathic diabetic subjects.

The altered patterns exhibited by neuropathic participants in adapting motor strategies when with or without shoes may also be present in other daily tasks. Therefore, an interesting way to improve the knowledge about the strategies adopted by diabetic neuropathic individuals is challenging their neuromuscular system by inducing voluntary increases in the cadence or performing other daily living activities, such as go up- or downstairs. A better understanding of the muscle activation pattern of neuropathic diabetic individuals during locomotor tasks is important for a suitable therapeutic intervention that aims for a better foot-floor interaction which can contribute to the prevention of plantar ulcerations.

## Conclusions

The use of shoes in diabetic neuropathic participants did not result in any delay of vastus lateralis activity compared with the non-diabetic participants, however there was a delay in the lateral gastrocnemius activation. The use of shoes did not reduce the vertical ground reaction forces at heel strike in diabetics and healthy controls. Furthermore, the results of the present study did not find any biomechanical data to support the notion that diabetic participants adjust their motor responses due to the use of shoes, although the use of shoes is highly recommended for diabetics to prevent distal lower limb injuries.

## Competing interests

The authors affirm that they have no financial affiliation (including research funding) or involvement with any commercial organization that has a direct financial interest in any matter included in this manuscript, except as disclosed in the attachment and cited in the manuscript.

## Authors' contributions

ICNS conceived the study, participated in its design and drafted the manuscript; PMHA participated in the conception of the study, in the data acquisition and analysis and helped to draft the manuscript; EWH participated in the conception of the study, helped to draft and final review of the manuscript. All authors read and approved the final manuscript.

## Pre-publication history

The pre-publication history for this paper can be accessed here:

http://www.biomedcentral.com/1471-2474/11/24/prepub
